# Dynamic Wavefront Manipulation Enabled with VO_2_-Based Reflective Terahertz Metasurfaces

**DOI:** 10.3390/nano16050338

**Published:** 2026-03-09

**Authors:** Ruifan Huang, Shangchu Shi, Mohan Sun, Rui Yang, Yizhen Lin, Mingzhong Wu, Mingze Zhang, Sergey Maksimenko, Xunjun He

**Affiliations:** 1 Department of Electronic Science and Technology, School of Electrical and Electronic Engineering, Harbin University of Science and Technology, Harbin 150080, China; 18860005268@163.com (R.H.); 18804610838@163.com (S.S.); christophergriffin6061@outlook.com (M.S.); 18804610991@163.com (R.Y.); 15860004897@163.com (Y.L.); 13965142314@126.com (M.W.); zhangingze@hrbust.edu.cn (M.Z.); 2 Research Institute for Nuclear Problems, Belarusian State University, 220030 Minsk, Belarus; maksim@bsu.by

**Keywords:** THz metasurface, wavefront, VO_2_, focused beam, vortex beam, dynamic control

## Abstract

Dynamic wavefront control plays a crucial role in advancing terahertz (THz) high-precision non-destructive testing, wireless communication and high-resolution imaging. However, existing approaches to THz dynamic wavefront control suffer from inherent limitations, such complex structures, narrow operational bandwidth, and the ability to tune only a single function, significantly restricting their practical applications. To overcome these challenges, we propose a dynamic reflective THz metasurface based on nested split-ring unit cells. The nested unit cell consists of an outer double-split VO_2_ ring resonator and an inner single-split aluminum ring deposited on a central VO_2_ circular patch. By, respectively, rotating the inner and outer rings in the insulator and metal states of VO_2_, independent full 2π phase coverage at 1.07 THz can be achieved in both VO_2_ states while maintaining high polarization-conversion efficiency with a PCR exceeding 0.98, thereby enabling efficient dynamic wavefront control. Using these unit cells, we constructed three distinct reflective metasurfaces that, respectively, generate broadband focusing beams with tunable focal lengths, broadband vortex beams with different topological charges, and a broadband beam that can be switched between focusing and vortex modes by changing the state of VO_2_. The design offers considerable flexibility for developing compact, multifunctional THz devices, with promising potential for integrated THz systems, high-capacity communications, and high-resolution imaging.

## 1. Introduction

With the rapid advancement of terahertz (THz) technology, THz wavefront control has garnered significant attention in cutting-edge fields including high-resolution imaging, biosensing, and non-destructive testing [1,2,3,4,5,6]. Currently, most approaches to THz wavefront control still rely primarily on conventional optical components such as lenses, prisms, spatial light modulators, and spiral phase plates [7,8,9]. However, these devices are often limited by large footprints and complex structure, which restrict their practical deployment and broader application [10,11,12,13]. In contrast, metasurfaces, as two-dimensional planar structures composed of subwavelength unit cells arranged in specific sequences, offer a promising alternative [14,15]. By carefully designing the geometry, size, and arrangement of these unit cells, metasurfaces enable precise control over the phase, amplitude, and polarization of incident waves [16,17,18]. These capabilities support the realization of diverse complex THz wavefront functions, such as planar focusing lenses, beam deflectors, and vortex beam generators [19,20]. Furthermore, recent years have seen metasurfaces expand beyond single wavefront manipulation toward multi-dimensional and intelligent applications, with substantial potential in nonreciprocal optical control and human–machine intelligent interaction, offering novel solutions for next-generation photonic systems and wireless communication networks [21,22].

To further enable dynamic THz wavefront manipulation, recently, metasurfaces integrated with functional materials have emerged as a novel platform. For example, Dai et al. demonstrated a 3-bit reconfigurable THz reflective metasurface using split-ring resonators embedded with photosensitive germanium, enabling the generation of vortex beams with different topological charges [23]. Chen et al. proposed a broadband polarization-independent THz multifunctional coding metasurface based on liquid crystal, which enables dynamic wavefront reconfiguration including beam steering and vortex beam generation [24]. Liu et al. realized a chiral THz metasurface utilizing graphene structure to achieve switchable focusing of circularly polarized waves [25]. Wang et al. reported a liquid-crystal-integrated THz metalens capable of switching between polarization-independent single focusing and spin-selective bifocal operation [26]. Li et al. proposed a new metasurface based on twin rectangular graphene patterns for generating vortex beams with tunable topological charges and focusing beams with tunable focal lengths [27]. Despite the remarkable progress in dynamic wavefront control, existing approaches still suffer from inherent limitations: complex architectures, slow response speeds, narrow operating bandwidths, and single-function modulation rather than true multifunctional reconfiguration. These drawbacks severely restrict their practical deployment in complex scenarios that demand versatile, broadband, and stable wavefront manipulation [28].

To address these challenges, we propose a reflective THz metasurface based on nested VO_2_–metal split-ring unit cells for active wavefront control. Each unit cell comprises an outer double-split VO_2_ ring resonator and an inner single-split aluminum ring resonator deposited on a central VO_2_ circular patch. By rotating the outer and inner rings corresponding to the insulating and metallic states of VO_2,_ respectively, independent full 2π phase coverage at 1.07 THz can be achieved in both states while preserving high polarization-conversion efficiency under left-handed circularly polarized (LCP) illumination. Based on this design, we constructed three functional metasurfaces operating across a broad frequency range, respectively, enabling different dynamic wavefronts: focusing beams with tunable focal lengths, vortex beams with varying topological charges, and dynamic switching between focusing and vortex beams. This approach provides a flexible strategy for developing miniaturized multifunctional THz wavefront devices, with promising potential for high-capacity 6G communications, high-resolution imaging, and integrated THz micromanipulation systems.

## 2. Designs and Simulations of Unit Cells

**Figure 1:**
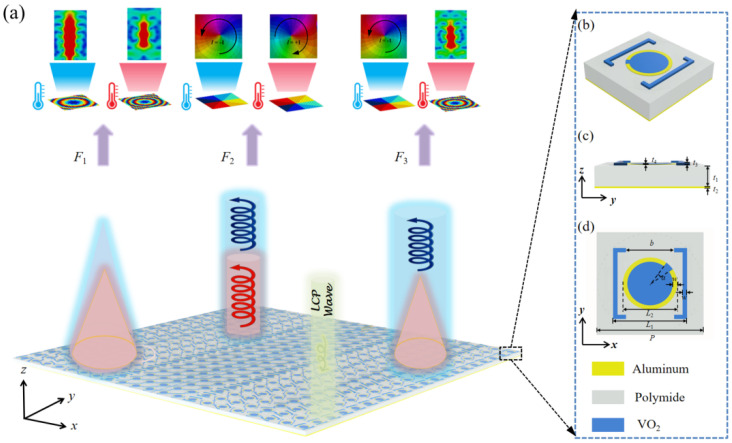
Schematic of the designed metasurface and unit cell: (**a**) functional illustration of the metasurface, (**b**) 3D perspective view, (**c**) side view, and (**d**) top view of unit cell.

To achieve dynamic wavefront control, as shown in Figure 1a, we designed a three-layer “metal-dielectric-metal” unit cell, as illuminated in Figure 1b–d. From top to bottom, the unit cell comprises a top layer of nested split-ring resonators, a polyimide dielectric layer, and an aluminum metal substrate. The top structure features an outer double-split VO_2_ ring resonator and an inner single-split aluminum ring deposited on a central VO_2_ circular patch. In the metallic state, the outer VO_2_ ring resonator acts as the main phase-shifting element, providing full 2π phase coverage within a wide frequency range for LCP incidence through structural rotation. In contrast, the inner aluminum resonator serves as the primary tuning component in the insulating state, also offering independent 2π phase coverage control via rotation, thereby enabling dynamic wavefront control within a broad frequency band. Additionally, the circular VO_2_ patch enhances the polarization-conversion efficiency in both operational states. Compared to the previously reported structures [29], our structure not only achieves phase reconfiguration over a broad bandwidth with high polarization-conversion efficiency, but also enables actively dynamic wavefront control. To verify the designed performance, full-wave simulations were conducted using the finite-difference time-domain (FDTD) solver in CST microwave studio. Periodic boundary conditions were applied in the *x*- and *y*-directions, while the Zmax and Zmin were set as an open boundary. An LCP wave was normally incident along the *z*-direction. The optimized geometric parameters are summarized in Table 1. The conductivity of aluminum was set to *σ* = 3.56 × 10^7^ S/m, and the dielectric constant of polyimide was *ε*_PI_ = 3.5. For the phase-change material VO_2_, the conductivity was set to *σ* = 2 × 10^5^ S/m in the metallic state and *σ* = 200 S/m in the insulating state [30,31].

**Figure 2:**
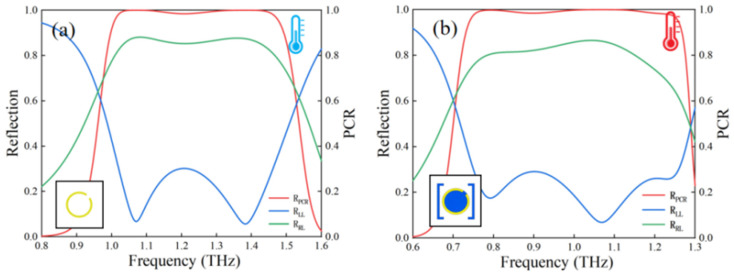
THz response of unit cell under LCP incidence: cross-polarization, co-polarization, and PCR at 1.07 for (**a**) insulating and (**b**) metallic states.

**Figure 3:**
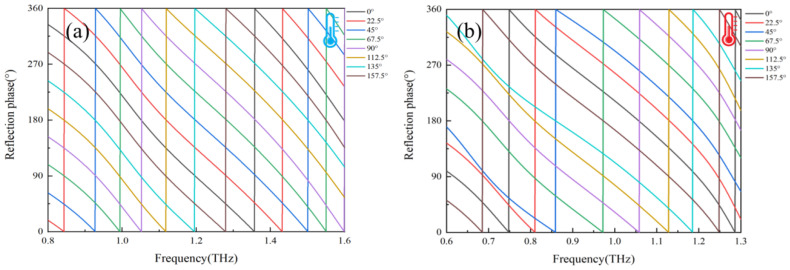
Cross-polarization phase of unit cells obtained by rotating different elements: (**a**) the inner ring in the insulating state and (**b**) outer ring in the metallic sate.

**Figure 4:**
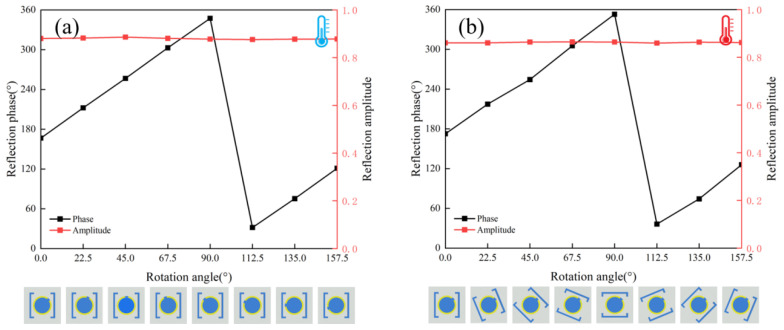
Amplitude and phase responses of the two sets of metasurface unit structures: (**a**) insulating state and (**b**) metallic state.

Figure 2a,b present the THz response of the unit cell under the insulating and metallic states of VO_2_, respectively. In the insulating state (Figure 2a), the cross-polarized reflection amplitude (*R*_RL_) exceeds 0.85 at 1.07 THz while the co-polarized reflection amplitude (*R*_LL_) remains below 0.25. The corresponding polarization conversion ratio (PCR) stays above 0.98 across the 1.0–1.4 THz band. Upon switching to the metallic state (Figure 2b), the *R*_RL_ remains over 0.8 at 1.07 THz with consistently low *R*_LL_, and the band with PCR > 0.98 shifts to 0.8–1.2 THz. Notably, the high-PCR regions of the two states overlap within the 1.0–1.2 THz range. Furthermore, by independently rotating the inner and outer rings in both VO_2_ states, full 2π phase coverage within this overlapping frequency band can be achieved for each state, as illustrated in Figure 3. Based on the aforementioned analysis, two sets of unit libraries were established, each comprising eight metasurface units. Distinct phase shifts were introduced by rotating the inner and outer rings on the top layer of the metasurface units, as illustrated in Figure 4. Together, these elements establish a crucial foundation for dynamic wavefront control.

**Figure 5:**
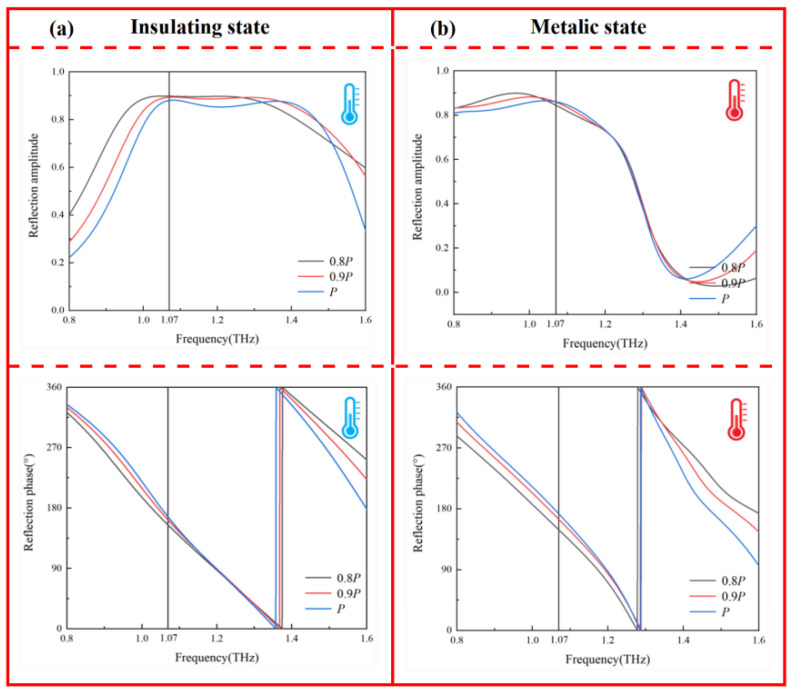
Reflection characteristics with varying unit period *P*: (**a**) insulating state and (**b**) metallic state.

**Figure 6:**
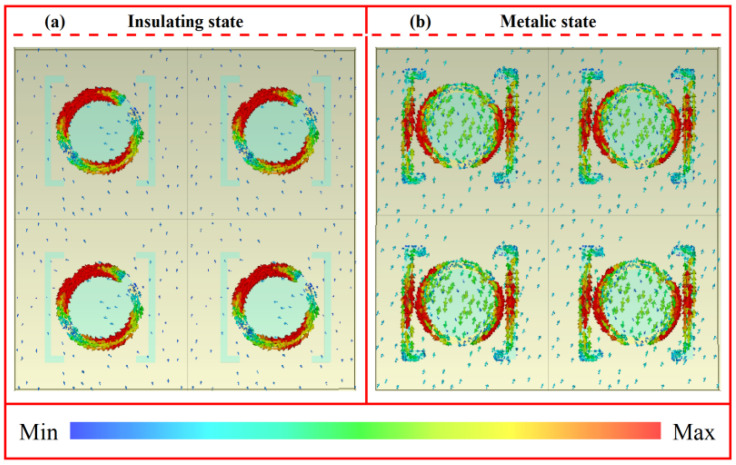
Surface current distribution at 1.07 THz: (**a**) insulating state and (**b**) metallic state.

To further evaluate the mutual coupling between adjacent unit cells, we analyzed the influence of the unit cell period on the phase and amplitude under both VO_2_ states. As illustrated in Figure 5, when the period P of the unit cell was reduced by 10–20% while keeping other parameters unchanged, the resulting variation in the phase and amplitude at 1.07 THz were negligible and can be disregarded. This stability indicates that the electromagnetic response of the unit cells is not governed by mutual inductive or capacitive coupling, thereby ensuring that the unit behavior in a complex coded array environment remains highly consistent with that of the isolated single-cell model. Moreover, Figure 6 shows the near-field distribution characteristics of the unit under left-handed circularly polarized (LCP) wave excitation. It can be clearly observed that under both VO_2_ states, the induced currents were tightly confined to the edge regions of the top single-split metal ring, the VO-based double-split rings, and the internal patch, while decaying rapidly and approaching zero near the unit’s periodic boundaries. This localization of electromagnetic energy within the physical boundaries of a single cell provided direct evidence for the absence of significant inter-unit energy transfer or electromagnetic crosstalk.

## 3. Dynamic Wavefront Control

### 3.1. Focus-Tunable Focusing Beams

**Figure 7:**
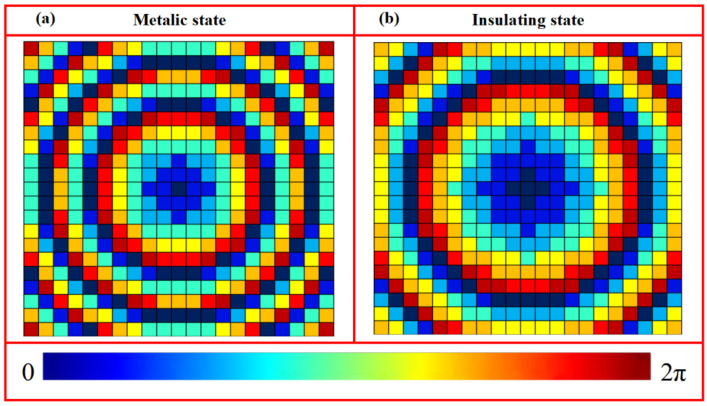
Phase distributions of metasurface composed of a 21 × 21 unit cells for tunable focal length: (**a**) *f*_I_ = 800 μm and (**b**) *f*_II_ = 1600 μm.

Varifocal metalenses are highly valuable for applications in THz imaging, sensing, and medical diagnostics, as they enable dynamic and adaptive wavefront control without bulky mechanical systems [27,32,33,34]. To implement a metalens whose focal length can be switched between $f_{I}$ and $f_{\mathrm{II}}$, the corresponding phase profiles must generally satisfy the following expressions [35]:

(1) $$\left\{\begin{array}{l} \varphi_{I}(x,y)=\frac{2\pi }{\lambda }(\sqrt{x^{2}+y^{2}+f_{I}^{2}}-f_{I})\\ \varphi_{\mathrm{II}}(x,y)=\frac{2\pi }{\lambda }(\sqrt{x^{2}+y^{2}+f_{\mathrm{II}}^{2}}-f_{\mathrm{II}}) \end{array}\right.$$

where *λ* denotes the incident wavelength, and (*x*, *y*) represent the coordinates of the unit cell center. To enable foal-length switching between *f*_I_ = 800 μm and *f*_II_ = 1600 μm at 1.07 THz using the metallic and insulating states of VO_2_, a reflective metasurface, composed of a 21 × 21 array of unit cells, was constructed based on a predefined library. The corresponding phase distributions derived from Equation (1) are illustrated in Figure 7.

**Figure 8:**
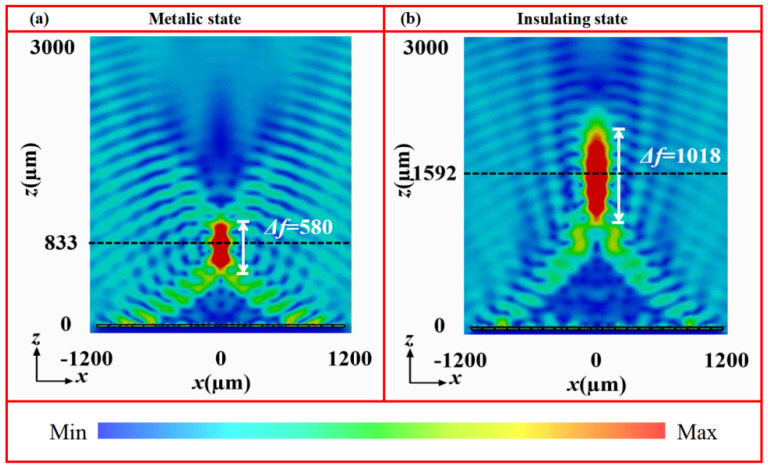
Electric field distributions of the focused metasurface at 1.07 THz under different states: (**a**) metallic state and (**b**) insulating state.

**Figure 9:**
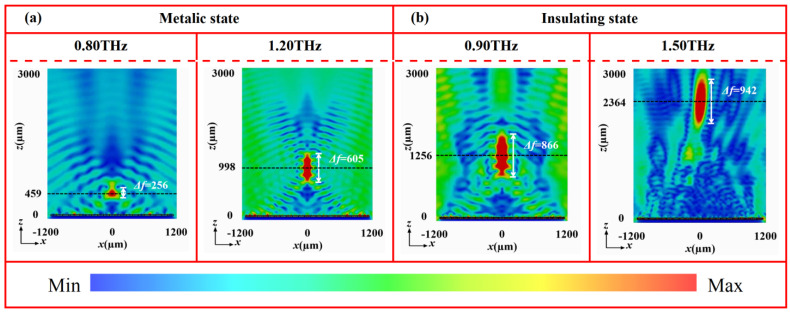
Electric field distribution of a focusing metasurface at different frequencies and states: (**a**) 0.80 THz and 1.20 THz at metallic state and (**b**) 0.90 THz and 1.50 THz at insulating state.

Figure 8 shows the electric field intensity distributions in the *x*-*z* plane of the tunable-focus metalens at 1.07 THz under both VO_2_ states. In the metallic state (Figure 8a), the reflection wave converges into a well-defined focal spot at *z* = 833 μm, with a focal depth of 580 μm. The focal position deviates only slightly from the designed value (*f*_I_ = 800 μm), which can be attributed to the finite size of in the metasurface array [36,37,38]. In contrast, when VO_2_ is the insulating state (Figure 8b), the focal spot shifts notably along the propagation direction, accompanied by an increase in the focal depth with longer focal length. Here, the energy concentrates distinctly at *z* = 1592 μm with a focal depth of 1018 μm, aligning closely with the preset focal length (*f*_II_ =1600 μm) with minimal error. These simulation results confirm that the tunable focusing behavior arises from the phase-change properties of VO_2_. Specifically, the distinct conductivity between its metallic and insulating states reconfigures the phase profile of the metasurface, thereby achieving effective dynamic wavefront control using a single device architecture. To further evaluate the broadband performance, the focusing capability of the metalens was examined across multiple frequencies. As shown in Figure 9, a well-defined focal spot is maintained from 0.80 to 1.20 THz in the metallic state. Similarly, clear focusing is observed from 0.90 to 1.50 THz in the insulating state. These results verify that this device possesses a broad operational bandwidth in both phase states, providing a reliable device-level strategy for dynamic wavefront control in a wide THz frequency range.

### 3.2. Topological Charge-Tunable Vortex Beams

**Figure 10:**
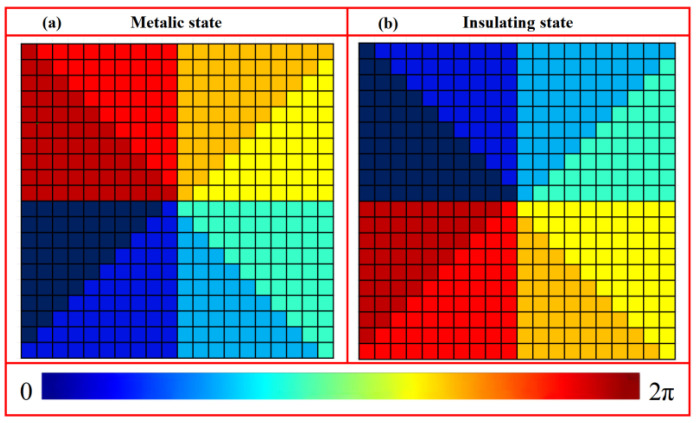
Phase distributions of metasurface for generating tunable vortex beams under different states: (**a**) metallic state and (**b**) insulating state.

Vortex beams, characterized by helical phase fronts and doughnut-shaped intensity profiles, carry orbital angular momentum (OAM) whose modes with different topological charges are inherently orthogonal [39,40]. This orthogonality prevents mutual interference between vortex beams with distinct topological charges, enabling simultaneous transmission of multiple beams within the same spatial channel. This mode-division multiplexing technique provides a powerful means to substantially increase the channel capacity and spectral efficiency of future communication systems, meeting the growing demand for high-throughput applications such as high-capacity communication, high-resolution imaging, and sensing [41,42]. To generate a vortex beam whose topological charge can be dynamically switched between specific values (*l*_I_ = +1 and *l*_II_ = +1), the corresponding phase distribution must generally satisfy the following expressions [43]:

(2) $$\left\{\begin{array}{c} \varphi_{I}(x,y)=l_{I}\;\arctan(y/x)\\ \varphi_{\mathrm{II}}(x,y)=-l_{\mathrm{II}}\;\arctan(y/x) \end{array}\right.$$

where *l* denotes the topological charge, and (*x*, *y*) are the coordinates of the unit cell center. Using both states of VO_2_, here, we designed a reflective metasurface consisting of a 20 × 20 array of unit cells selected from the predefined library to achieve a vortex beam with a switchable topological charge. This configuration enables the switching between *l* = +1 and *l* = −1 at 1.07 THz. The corresponding phase distributions derived from Equation (2) are presented in Figure 10.

**Figure 11:**
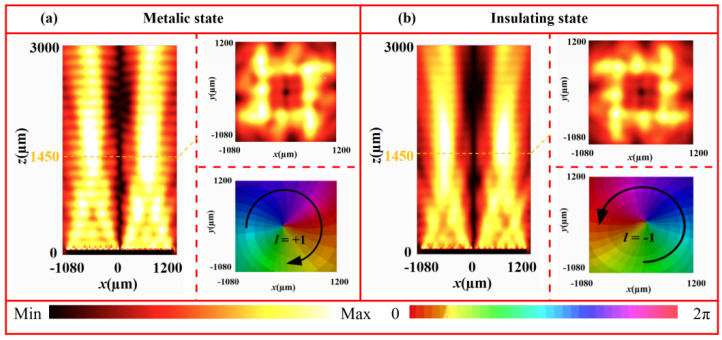
Electric field intensity and phase distributions of vortex beams at 1.07 THz under different states: (**a**) metallic and (**b**) insulating states.

**Figure 12:**
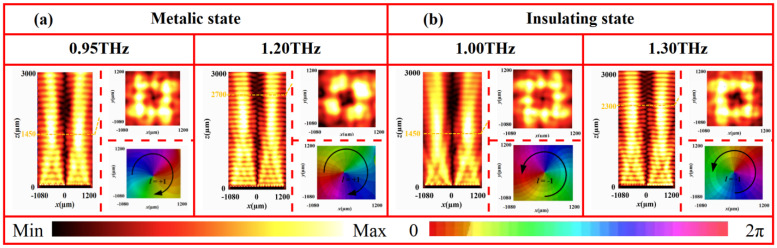
Electric field and phase distributions of vortex beams at different frequencies and states: (**a**) 0.95 THz and 1.20 THz at metallic state and (**b**) 1.00 THz and 1.30 THz at insulating state.

Figure 11 presents the electric field intensity distributions (in the *x*-*z* and *x*-*y* planes) and phase profiles of the generated vortex beams at 1.07 THz for both VO_2_ states. In the metallic state (Figure 11a), the electric field intensity in the *x*-*y* plane (at *z* = 1450 μm) exhibits a characteristic annular pattern with the near-zero-intensity center. In the *x*-*z* plane, the annular radius expands progressively along the propagation direction without significant distortion. The corresponding phase profile shows a complete clockwise 2π spiral, confirming the successful generation of a vortex beam with topological change *l* = +1. Conversely, in the insulating state (Figure 11b), the electric field in the *x*-*y* plane (at *z* = 1450 μm) likewise displays a clear annular distribution with a central null. In the *x*-*z* plane, the reflective wave propagates helically along the axis, with a gradually expanding ring radius. The phase profile exhibits a distinct counter-clockwise 2π spiral, matching the characteristics of vortex beam with *l* = −1. These results demonstrate that the designed metasurface can generate a vortex beam with switchable topological charge by utilizing both states of VO_2_. This tunability originates from the phase transition of VO_2_. The change in conductivity between its metallic and insulating states effectively reconfigures the phase distribution of the metasurface, thereby enabling dynamic wavefront control within a single device. To evaluate the broadband performance, vortex beam generation was further examined at multiple frequencies. As shown in Figure 12, in the metallic state, vortex beams with *l* = +1 are stably produced from 0.95 to 1.20 THz. Similarly, in the insulating state, vortex beams with *l* = −1 are consistently generated from 1.00 to 1.30 THz. These findings confirm the excellent broadband vortex-generation capability of the metasurface in both phase states.

### 3.3. Dynamic Switching Between Focusing and Vortex Beams

**Figure 13:**
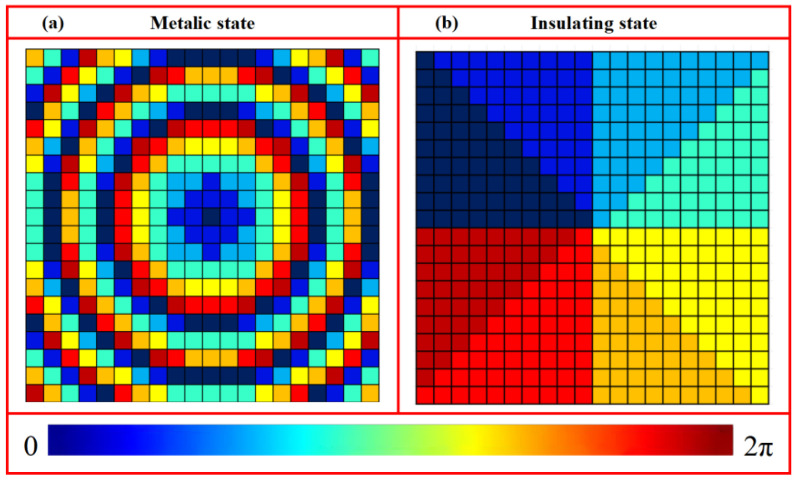
Phase distributions of metasurface for switching between focused beam and vortex beams under different VO_2_ states: (**a**) metallic state and (**b**) insulating state.

The energy-focusing property of focused beams and the orthogonality of OAM carried by vortex beams are fundamental enablers for key THz technology applications, including dynamic micromanipulation, high-capacity communication, and high-resolution imaging [44,45]. To achieve dynamic switching between a focused beam and a vortex beam, the required phase profiles are typically described by the following expressions:

(3) $$\left\{\begin{array}{l} \varphi_{f}(x,y)=\frac{2\pi }{\lambda }(\sqrt{x^{2}+y^{2}+f^{2}}-f)\\ \varphi_{l}(x,y)=l\;\arctan(y/x) \end{array}\right.$$

where λ is the incident wavelength, *l* is the topological charge, and (*x*, *y*) denote the coordinates of the unit cell center. To enable functional switching between a metalens with a focal length of *f* = 800 μm and a vortex beam with *l* = −1 at 1.07 THz by exploiting the metallic and insulating states of VO_2_, we designed a reflective metasurface composed of a 20 × 20 array of unit cells selected from a predefined library. The corresponding phase distributions calculated from Equation (3) are shown in Figure 13.

**Figure 14:**
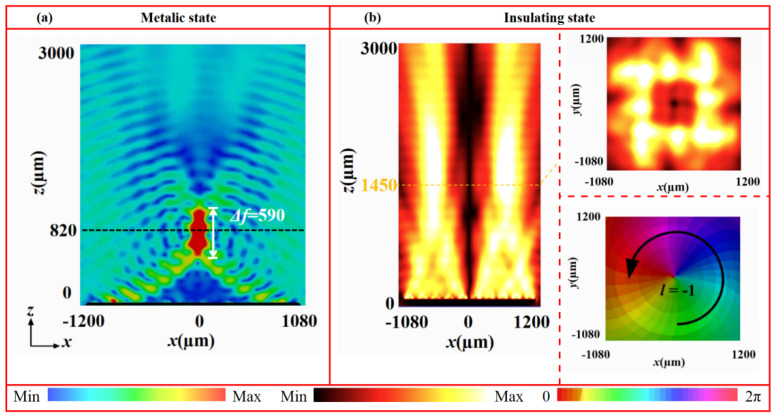
Electric field and phase distributions of focusing/vortex-switchable metasurface at 1.07 THz under different states: (**a**) metallic state and (**b**) insulating state.

**Figure 15:**
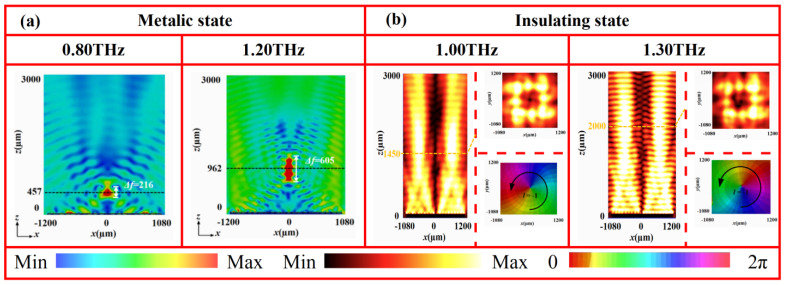
Electric field and phase distributions of focusing/vortex-switchable metasurface at different frequencies and states: (**a**) 0.80 THz and 1.20 THz at metallic state and (**b**) 1.00 THz and 1.30 THz at insulating state.

Figure 14 shows the simulated electric field intensity and phase distributions of the metasurface at 1.07 THz under different VO_2_ states. When VO_2_ is in the metallic state (Figure 14a), the reflection wave converges into a well-defined focal spot at *z* = 820 μm, with the focal depth of approximately 590 μm. In contrast, when VO_2_ is in the insulating state (Figure 14b), the electric field in the *x*-*y* plane (*z* = 1450 μm) displays a typical annular pattern with zero intensity at the center. In the *x*-*z* plane, the field maintains a V-shaped profile along the propagation axis, with the ring radius expanding gradually. Moreover, the corresponding phase profile exhibits a distinct counter-clockwise 2π spiral, confirming the generation of a vortex beam with topological charge *l* = −1. These results verify that dynamic switching between a focusing metalens and a vortex beam generator can be realized by exploiting the phase transition of VO_2_. To further access the broadband performance of this reconfigurable device, the focusing and vortex-generation capabilities were examined across multiple frequencies. As shown in Figure 15, in the metallic state, a clear focal spot is maintained from 0.80 to 1.20 THz. In the insulating state, vortex beams with *l* = −1 are stably generated from 1.00 to 1.30 THz. This confirms the broadband adaptability and functional robustness of the device across both phase states. To further highlight the novelty and advantages of the proposed design over previous works, a concise comparison is summarized in Table 2. The comparison clearly shows that the proposed design outperforms previously reported THz devices in several key aspects, including wider operational bandwidth, reduced structural complexity (fewer layers), and enhanced multifunctional switching capability.

## 4. Potential Manufacturing Processes

**Figure 16:**
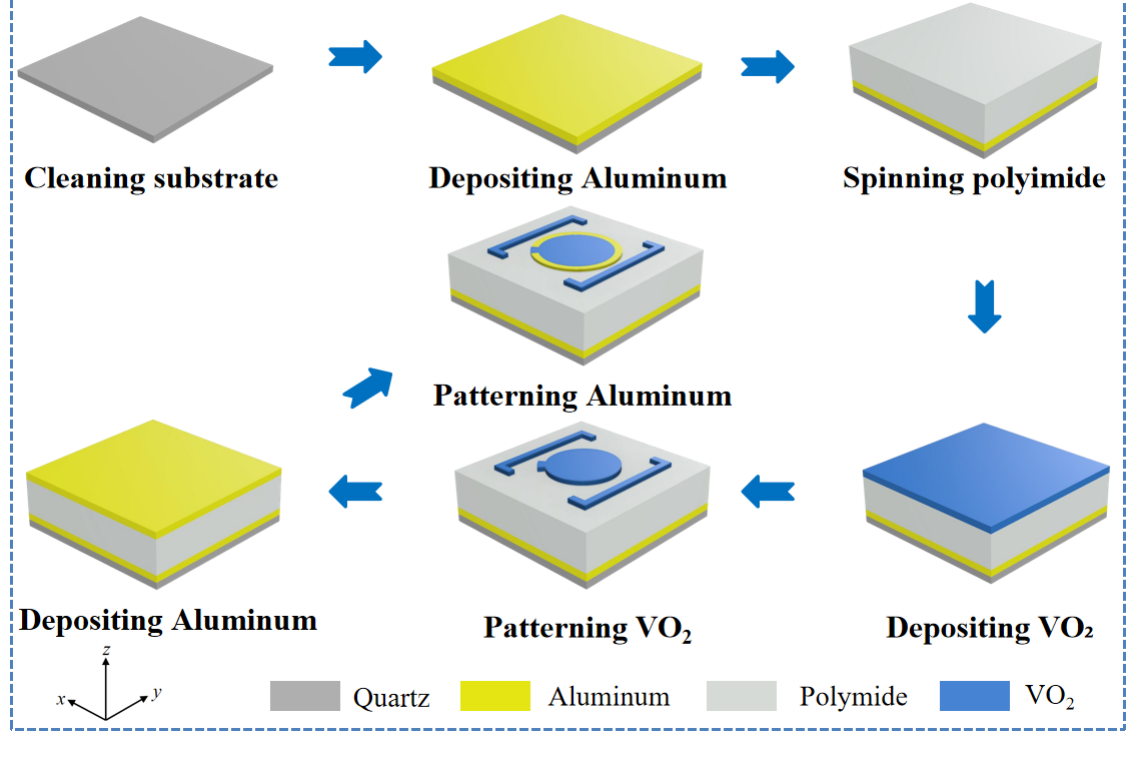
Manufacturing process of reflective THz metasurface based on VO_2_.

The fabrication process for the proposed metasurfaces is illustrated in Figure 16. First, the quartz wafer is cleaned sequentially with acetone, ethanol, and de-ionized water. An aluminum film is then deposited onto the cleaned substrate via magnetron sputtering. Subsequently, a polyimide layer is spin-coated onto the aluminum film and cured at elevated temperatures to form a uniform film. A VO_2_ film is then deposited by magnetron sputtering, followed by low-temperature annealing to improve crystallinity and phase-transition performance. Photoresist is spin-coated onto the VO_2_ layer, patterned using photolithography, and the VO_2_ film is etched into the desired structures via reactive ion etching. Finally, a top aluminum film is deposited by magnetron sputtering, followed by spin-coating of photoresists and pattering using conventional ultraviolet photolithography to achieve top metallic single-split-ring resonators.

## 5. Conclusions

In summary, we have successfully demonstrated a reconfigurable reflective THz metasurface for dynamic wavefront control based on a nested split-ring unit cell array. The unit cell comprises an outer double-split VO_2_ ring resonator and an inner single-split aluminum ring resonator deposited on a central VO_2_ circular patch. By, respectively, rotating these rings in the insulating and metallic states of VO_2_, independent full 2π phase coverage can be achieved at 1.07 THz in both VO_2_ states while maintaining high polarization-conversion efficiency with a PCR exceeding 0.98. Using these unit cells, three distinct functional metasurfaces were designed to implement varifocal metalens, a vortex generator with tunable topological charge, and a wavefront-switchable converter that toggles between focusing and vortex-generation modes. By adjusting the insulating and metallic states of VO_2_, the metalens enables the focal-length switching between *f*_I_ = 833 μm and *f*_II_ = 1592 μm at 1.07 THz. The vortex-generator produces vortex beams with switchable topological charges of *l* = +1 and *l* = −1, and the converter achieves dynamic switching between a focusing mode (*f* = 820 μm) and a vortex-generating mode (*l* = −1) at the same operating frequency. Moreover, these devices exhibit broadband operation, and simulated results agree closely with design expectations. Therefore, our design provides a highly flexible platform for developing compact, multifunctional THz devices, with strong potential for applications in integrated THz systems, high-capacity communications, and high-resolution imaging.

## Figures and Tables

**Table 1:** Optimized geometric parameters of unit cells.

Parameters	Values (μm)	Parameters	Values (μm)
*P*	120	*α*	20
*L*_1_	83	*t*_1_	25
*L*_2_	61	*t*_2_	0.2
*w*	5	*t*_3_	3.0
*b*	54	*t*_4_	0.5

**Table 2:** Comparison between the proposed work and previously reported THz works.

Ref.	Functional Materials	Band Width(THz)	Number of Layers	Functional Switching
[23]	Ge	0.20	3	Vortex beam and holographic imaging
[46]	GST	0.37	3	None
[47]	Graphene	0.25	4	None
[25]	Graphene	0.35	4	None
[26]	LC	0.40	7	None
This work	VO_2_	0.70	3	Vortex beam and focusing beam

## Data Availability

Data are contained within the article.
